# Direct Measurement of Dissolved Gas Using a Tapered Single-Mode Silica Fiber

**DOI:** 10.3390/s24103200

**Published:** 2024-05-17

**Authors:** Panpan Sun, Mengpeng Hu, Licai Zhu, Hui Zhang, Jinguang Lv, Yu Liu, Jingqiu Liang, Qiang Wang

**Affiliations:** 1 Key Laboratory of Advanced Manufacturing for Optical Systems (CAS), Changchun Institute of Optics, Fine Mechanics and Physics, Chinese Academy of Sciences, Changchun 130033, China; sunpanpan21@mails.ucas.ac.cn (P.S.); humengpeng19@mails.ucas.ac.cn (M.H.); zhanghui195@mails.ucas.ac.cn (H.Z.); lvjg@ciomp.ac.cn (J.L.); liuyu@sklao.ac.cn (Y.L.); liangjq@ciomp.ac.cn (J.L.); 2 University of Chinese Academy of Sciences, Beijing 100049, China; zhulicai20@mails.ucas.ac.cn; 3 State Key Laboratory of Luminescence Science and Technology (SKLST), Changchun Institute of Optics, Fine Mechanics and Physics, Chinese Academy of Sciences, Changchun 130033, China

**Keywords:** tapered single-mode silica fiber, evanescent wave, dissolved gas, in-situ detection, direct absorption spectroscopy

## Abstract

Dissolved gases in the aquatic environment are critical to understanding the population of aquatic organisms and the ocean. Currently, laser absorption techniques based on membrane separation technology have made great strides in dissolved gas detection. However, the prolonged water–gas separation time of permeable membranes remains a key obstacle to the efficiency of dissolved gas analysis. To mitigate these limitations, we demonstrated direct measurement of dissolved gas using the evanescent-wave absorption spectroscopy of a tapered silica micro-fiber. It enhanced the analysis efficiency of dissolved gases without water–gas separation or sample preparation. The feasibility of this sensor for direct measurement of dissolved gases was verified by taking the detection of dissolved ammonia as an example. With a sensing length of 5 mm and a consumption of ~50 µL, this sensor achieves a system response time of ~11 min and a minimum detection limit (MDL) of 0.015%. Possible strategies are discussed for further performance improvement in in-situ applications requiring fast and highly sensitive dissolved gas sensing.

## 1. Introduction

Variations in dissolved gases in aquatic environments, such as oceans and lakes, have a significant impact on the organisms in the corresponding ecosystems [1]. For example, dissolved oxygen is essential for marine aerobes, and the scientific community predicted decreased dissolved oxygen in oceans in the next century [2,3]. Too much dissolved CO_2_ in the ocean could cause increased acidity, the destruction of coral reefs, or the significant expansion of anoxic dead zones [4]. Excessive dissolved ammonia would lead to the rapid growth of algae and the eutrophication phenomenon, and then the reduction of dissolved oxygen would deteriorate the fragile aquatic ecosystem [5]. Therefore, large-scale direct in-situ sensing is needed to qualitatively detect dissolved gas concentrations in aquatic environments and expand the spatial-resolution data set.

Recently, in situ detections have employed different technologies, such as membrane separation [6,7], semiconductor sensing [8,9], mass spectrometry [10,11], and infrared absorption spectroscopy [12,13,14,15], to determine the dissolved gas concentration in aquatic environments. Infrared absorption spectroscopy technology stands out over others due to its high sensitivity and specific absorption selectivity for different molecules of interest. Current absorption-spectroscopy-based methods that are suitable for in-situ dissolved gas measurement can be roughly divided into (1) direct absorption—tunable diode laser absorption spectroscopy [16,17,18,19] and cavity enhanced spectroscopy [20,21,22,23], and (2) indirect absorption—photoacoustic spectroscopy [15,24]. Usually, the realization of these technologies requires the special design of a water–gas separation device to extract dissolved gas in its gaseous phase. Hydrophobic and permeable membranes are commonly used in dissolved gas extraction. However, a limited gas separation efficiency of tens of microliters per minute often restricts the response time of the entire sensor [25]. Many efforts were carried out to shorten the response time by minimizing the size of the gas chamber [24] or using exponential fitting [26]. Even so, the response time of hours still hampers the measurement efficiency. Therefore, for further efficient investigation on a larger scale, it is crucial to achieve in situ dissolved gas measurement with fast response and easy implementation while inheriting the advantages of classic absorption spectroscopy methods.

As an exquisite platform for enhanced light–matter interaction, micro-nano fiber has aroused widespread interest in spectroscopic analysis, owing to its remarkable corrosion resistance, intricate microstructure, effortless installation, immunity to electromagnetic interference, minimal attenuation over long distances, and commendable biochemical compatibility. Paul and Kychakoff, as early as 1987, presented a comprehensive theoretical model, establishing the fundamental framework for the advancement of fiber evanescent-wave sensors [27]. In the same year, Hideo Tai demonstrated the application of tapered fiber in methane sensing by thermally and mechanically stretching a multimode fiber with a diameter of 125 µm, achieving a sensitivity of 1% [28]. In 1999, Mizaikoff pioneered the development of a mid-infrared fiber evanescent-wave spectrometer that utilizes the selective absorption of fiber evanescent waves. This instrument was primarily employed for quantifying hydrocarbons in aqueous environments and enabling underwater operation down to a depth of 300 m [29]. In 2004, Yoesf Raichlin et al. utilized a broadband light source combined with silver halide mid-infrared fiber to measure triphosphorous and dichlorvos in water, achieving a measurement accuracy of 1 ppm [30]. In 2012, Luzinova et al. used mid-infrared (MIR) evanescent field absorption spectroscopy to quantitatively analyze trace oils in water to achieve detection limits at ppm concentration levels. After that, the surface of the fiber was modified by grafted epoxidized polybutadiene layers to directly detect crude oil in a deionized water matrix with a ppb concentration level [31]. In 2013, Lu et al. utilized a polycrystalline silver halide sensor fiber that was modified with an ethylene/propylene copolymer membrane to achieve the detection of hydrocarbons in water. High detection sensitivity was obtained for CHC mixtures over a wide concentration range from 5 to 700 ppb [32]. In 2018, Yang et al. utilized Ge_20_Se_60_Te_20_ glass-tapered fibers for in situ detection of mixtures of methanol and dichloromethane through optical fiber evanescent-wave spectroscopy. The increase in sensitivity was verified by increasing the evanescent-wave signal in tapering fiber [33]. In 2021, Qi et al. presented a gas–liquid sensor based on chalcogenide-etched-tapered fiber functionalized with graphene oxide film. The sensors could detect VOCs such as formaldehyde and butane in the mid-infrared band, and the detection sensitivity of both solution and gas was 4.5091 a.u./mg∙mL^−1^ and 0.4812 a.u./vol.%, respectively [34]. In recent years, the advancement of optical fiber evanescent-wave sensing technology led to the emergence of various novel sensing structures with exceptional performance, which found extensive applications in environmental sewage monitoring, atmospheric monitoring, and the analysis of volatile organic compounds in seawater [35,36,37,38].

For most in situ dissolved gas sensing technologies of evanescent wave, the optical waveguide usually consists of a sensitive film with selective permeability. When the light wave propagates in the waveguide material under full reflection conditions, an evanescent-wave field is formed in the film and interacts with the gas dissolved within it. Although this reduces the response time of the system to some extent by directly measuring the sensitive film without completely separating water and gas, the material characteristics and permeability of the film seriously restrict both the response time and sensitivity of the sensing system [39]. Moreover, some interesting research focused on the mid-infrared spectral range to further explore the much higher absorption ability, utilizing mid-infrared devices as laser sources and sensing fibers. This strategy not only escalates the expenses associated with experimentation but also hampers the experimental process due to the inherent drawbacks of mid-infrared fiber, such as its relatively inferior rigidity and susceptibility to fracture.

In this study, we develop a straightforward and efficient dissolved gas sensing system in the near-infrared wavelength range utilizing a single-mode fiber evanescent wave combined with microfluidic technology. The direct absorption spectroscopy (DAS) method for analyte detection enables accurate monitoring of dissolved gas in water without the need for sensitization treatment. The feasibility of our approach is demonstrated by detecting aqueous ammonia samples, and the concentration of aqueous ammonia is determined through integrated absorbance. The experimental results show a response time of approximately 11 min, offering a simple and effective solution to enhance the response speed of in situ detection systems for dissolved gas in water.

## 2. Methods

### 2.1. Evanescent-Wave Dissolved Gas Sensing

**Figure 1:**
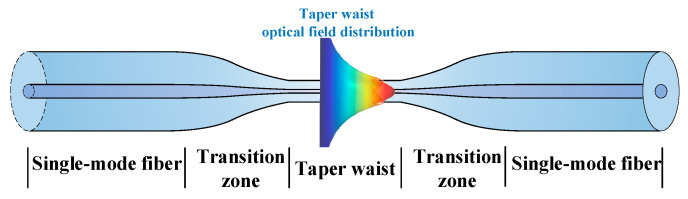
Schematic of the fused taper fabricated by oxyhydrogen flame for evanescent-wave generation.

When light propagates in the optical fiber by total reflection, partial energy flow, in the form of an evanescent wave, exists in the cladding. If the fiber cladding comprises a lossy medium, then the evanescent-wave energy flow could be partially absorbed, imprinting such information on the transmitted light waves. Replacing the cladding with analytes of interest enables convenient evanescent-wave sensing at the fiber end after collecting the transmitted photons. Its effective proportion of the total light power can be adjusted by controlling the fiber shape and size of the sensing area [40]. Spectral information is also accessible by the employment of a wavelength-tunable laser [41] or by the synergy of a broadband light source and a spectrometer [42]. A fused taper can be fabricated by a hydrogen–oxygen flame with the schematic shown in Figure 1. After proper fabrication, it can be divided into three distinct sections: the untapered segment (single-mode fiber), the tapered transition region (transition zone), and the beam waist (taper waist). With this structure, the fundamental mode of the untapered section is able to traverse the transition region and transform into the cladding mode at the beam waist.

When light at wavelength *λ* enters the fiber, the evanescent wave interacts with the analyte along the optical fiber surface. The interaction still obeys the classical Beer–Lambert law that relates the lights before and after light–matter interaction [43]. The evanescent-wave absorption coefficient (*γ*) can be equivalent as follows [27]:

(1) $$\gamma =\left(1-\eta \right)\alpha_{\mathrm{fiber}}+\eta \alpha_{\mathrm{analyte}}$$

where *η* represents the percentage of total power carried by the evanescent wave outside the optical fiber, *α*_fiber_ is the fiber’s absorption coefficient, and *α*_analyte_ is the analyte’s absorption coefficient. Due to the fact that, apart from the sensing area, the optical fiber cladding is replaced by the analyte, the material of the optical fiber remains an ideal homogeneous medium with almost no absorption loss, i.e., *α*_fiber_ ≈ 0. Although only the evanescent wave interacts with the analyte, the loss caused by analyte absorption is manifested in the overall light intensity, mainly due to the parasitic relationship between the evanescent wave and the light trapped in the fiber. Therefore, the power transmitted by an optical fiber whose cladding has been replaced locally by an absorbing medium may be written in the following form [44]:

(2) $$P_{1}=P_{0}\exp\left(-\gamma L\right)=P_{0}\exp\left(-\eta \alpha_{analyte}L\right)$$

where *P*_0_ is the output power of the light source without analyte absorption, *P*_1_ is the output power collected from the outgoing fiber end after absorption of the analyte, and *L* is the effective fiber length of the evanescent-wave absorption unit.

For liquid analytes, the refractive index *n*_2_ of the analyte to be measured is often much smaller than the core refractive index *n*_1_, and Δ = (*n*_1_ − *n*_2_)/*n*_1_≪1 will no longer be maintained. Hence, the optical fiber transitions from weak-guided mode in the transmission segment to strong-guided mode in the evanescent-wave absorption unit. The expression of the evanescent-wave absorption coefficient *γ* will be modified to the following [45]:

(3) $$\gamma =\frac{\lambda n_{2}\cos^{2}\theta }{2\rho \pi \left(n_{1}^{2}-n_{2}^{2}\right)\sin\theta \sqrt{\sin^{2}\theta -{\left(n_{2}/n_{1}\right)}^{2}}}\cdot \alpha$$

where *ρ* is the core radius, *θ* is the incident angle, *α* = *ε*∙*C* is the absorption coefficient of the liquid analytes, *ε* is the molar absorptivity of the analyte, and *C* is the concentration of the analyte to be measured. By substituting Equation (3) into Equation (2), we obtain the light energy transmission after the evanescent-wave absorption unit as follows:

(4) $$P_{1}=P_{0}\exp\left(-\frac{\varepsilon \lambda n_{2}\cos^{2}\theta }{2\rho \pi \left(n_{1}^{2}-n_{2}^{2}\right)\sin\theta \sqrt{\sin^{2}\theta -{\left(n_{2}/n_{1}\right)}^{2}}}CL\right)$$

According to Equation (4), with the incident angle, taper waist length, taper waist radius, incident wavelength, core refractive index, analyte refractive index, and its molar absorptivity held constant, the power of the optical fiber output end only depends on the concentration of the target liquid. Consequently, changes in the concentration level of the target liquid can be detected by monitoring variations in either the optical power or spectrum at the output end of the optical fiber.

The absorbance that determines the concentration of the analyte can be given by the following:

(5) $$A=-\log_{10}\left(\frac{P_{1}\left(\lambda \right)}{P_{0}\left(\lambda \right)}\right)$$

In the spectrum of the evanescent-wave field, in addition to absorption, light waves also undergo attenuated total internal reflection and frustrated total reflection near the critical angle. This makes it challenging to determine concentration solely by analyzing line shape and width. Thus, for direct absorption spectroscopy, the concentration can also be determined by integral absorbance [46], mitigating potential noise from intensity jitter or optical interference. The integral absorbance for evanescent-wave absorption, *A*_integral_, can be defined as follows:

(6) $$A_{\mathrm{integral}}=\int -\log_{10}\left(\frac{P_{1}\left(\lambda \right)}{P_{0}\left(\lambda \right)}\right)d\lambda$$

### 2.2. Micro-Fiber and Micro-Channel Preparation

**Figure 2:**
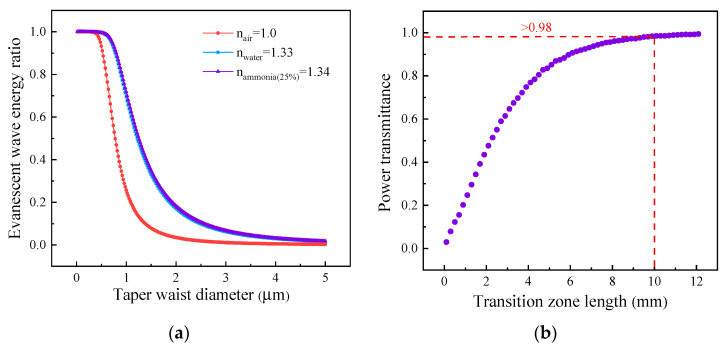
(**a**) Evanescent-wave power ratio at the taper waist calculated in air, pure water, and aqueous ammonia (25% in concentration) as a function of diameter. (**b**) The relationship between power transmittance and transition zone length.

A piece of fused tapered fiber was used as the micro-fiber, and its preparation was performed by the hydrogen–oxygen flame brush method, which has the advantages of easy preparation, a smooth surface, morphology control, and high transmittance. As shown in Figure 2a, we utilized the wave optics module in COMSOL Multiphysics to simulate the variation trend of evanescent-wave intensity with diameter in air, pure water, and aqueous ammonia (25% in concentration), respectively, at 1550 nm. The intensity of the evanescent wave increases as the waist diameter decreases or as the external refractive index approaches that of the fiber. Additionally, we assessed the correlation between power transmittance and taper length while maintaining a constant taper waist of 2 µm (Figure 2b). The power transmittance increases with an elongation of the transition zone length. For short transition zones with a sharper slope, it is easier for the fundamental mode to couple into higher-order modes. Conversely, only a single mode or a limited number of low-order modes are permissible in the waist region, leading to additional power dissipation. Therefore, achieving a longer transition region by gradually reducing its diameter ensures higher transmittance, and theoretically, a transition zone length of 10 mm guarantees a power transmittance exceeding 98%.

**Figure 3:**
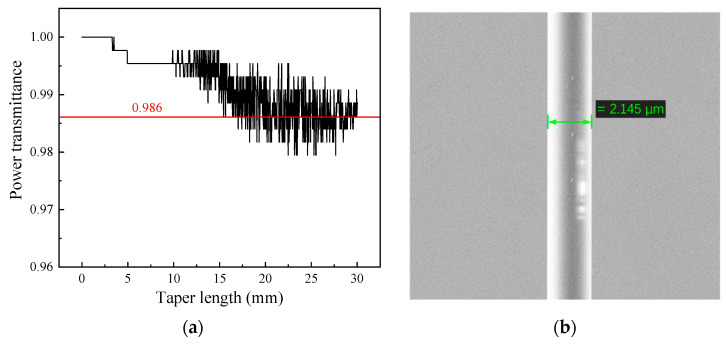
(**a**) Monitored real-time power transmittance of the tapered fiber during the fiber drawing process. (**b**) Scanning electron microscope (SEM) image of the fabricated micro-fiber.

Therefore, as a tradeoff between mechanical stability and proportion of evanescent wave, we conducted parameter optimization on the optical fiber diameter, sensing length, and transition zone interaction (waist diameter: 2.0 µm; waist length: approximately 5 mm; transition zone length: 12.5 mm) [47] for the fabrication of a tapered micro-fiber. Figure 3a shows the monitored real-time power transmittance of the tapered fiber during the fiber drawing process, which reaches a final plateau level of 98.6% (~0.06 dB). Meanwhile, the surface morphology of the micro-fiber was examined using a scanning electron microscope (SEM), as depicted in Figure 3b, showing a diameter of ~2.145 µm. The refractive index of aqueous ammonia in the near infrared region is slightly higher than that of water and changes little with an increase in ammonia concentration. The calculated evanescent waves generated by the micro-fibers with this diameter are 14.6% and 15.5%, respectively, yielding a small difference of ~0.9%. This difference would be further reduced with the normalization process. Therefore, the influence of the difference in evanescent-wave intensity between aqueous ammonia and pure water on the overall experimental results is disregarded for the sake of convenience in calculation, given the little change in refractive index.

**Figure 4:**
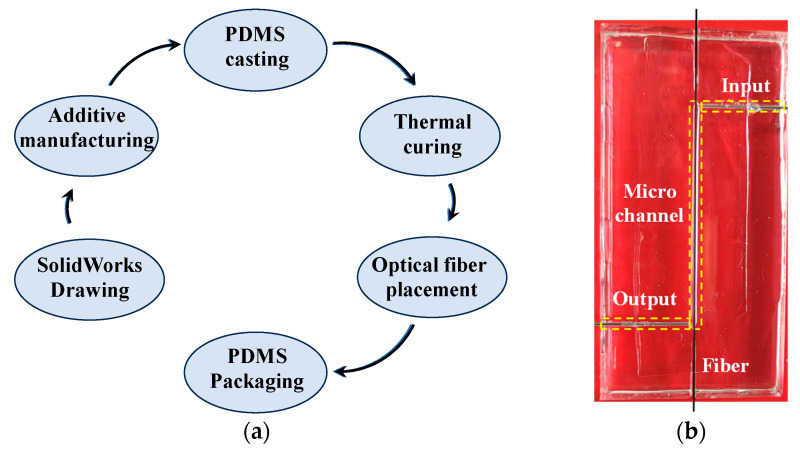
(**a**) Flowchart depicting the fabrication process of a microchannel device. (**b**) Photo of a microchannel device.

In this study, the organic polymer material polydimethylsiloxane (PDMS) from DOWSIL (DC184) was utilized to fabricate microfluidic channels and integrate with micro-fibers. The specific fabrication process is illustrated in Figure 4a. We initially fabricated a microchannel model (80 × 40 × 10 mm^3^) using additive manufacturing technology with transparent photosensitive resin. Considering the limitations of additive manufacturing in achieving minimum bulge size and the simplicity required for micro-fiber packaging, we set the bump width and height of the model to be 0.8 mm and 1.5 mm, respectively, resulting in a liquid volume of only ~50 µL for reducing sample consumption. Subsequently, polydimethylsiloxane material was injected into the model and heated up to 80 °C for two hours in an oven. After the curing process, the PDMS material was extracted from the mold and positioned onto a three-dimensional displacement assembly platform. Subsequently, we precisely arranged the micro-fiber within the microchannels, which were then hermetically sealed using a gas-tight PDMS film, as shown in Figure 4b.

## 3. Experimental Setup

**Figure 5:**
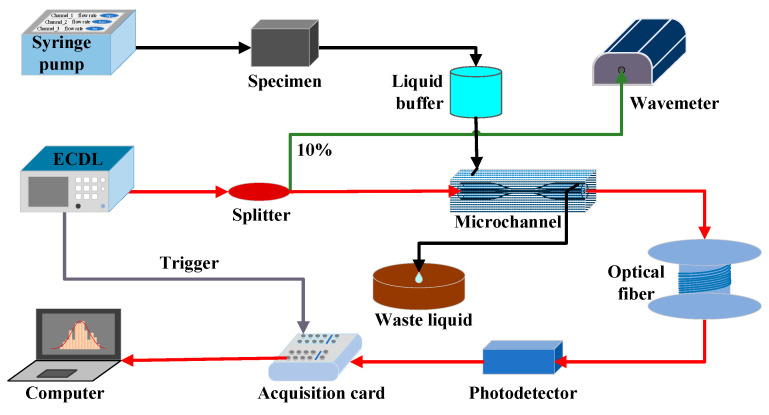
Schematic diagram of the experimental setup.

Figure 5 depicts a schematic diagram of an experimental setup used to directly detect aqueous ammonia with a silica micro-optical fiber. The micro-fiber was fabricated by using hydrogen flame to stretch silica single-mode fiber (SMF-28E+, Corning, Corning, NY, USA) with controllable waist diameter and length [47]. The micro-fiber was encapsulated inside the PDMS microchannel, which can be filled with a liquid sample of aqueous ammonia with a controllable concentration as needed for sensing calibration. An external-cavity diode laser (TSL-550, Santec, Komaki, Japan) is connected to one end of the micro-fiber through a splitter (90:10) that separates a small portion of light into a wavemeter (671B, Bristol, UK) for monitoring purposes. The laser wavelength, from 1480 nm to 1630 nm, scans the sample in the form of a sawtooth wave. Finally, a photodetector (DET08CFC/M, ThorLabs, Newton, NJ, USA) detects the transmitted light intensity, which is digitized to a high-speed data acquisition card (PCI-6356, National Instruments, Austin, TX, USA) for collection and processing. Inspired by commonly used medical transfusions, a liquid buffer (550 µL) is used to prevent bubbles from entering and leaving the microchannel during the sample-changing process. A ~3-kilometer-long fiber spool serves primarily as a filtering mechanism to eliminate residual high-order modes that may persist after transmission through the micro-optical fiber, thereby mitigating potential interference fringes.

## 4. Experimental Results and Discussion

### 4.1. Absorption Spectroscopy of Aqueous Ammonia

**Figure 6:**
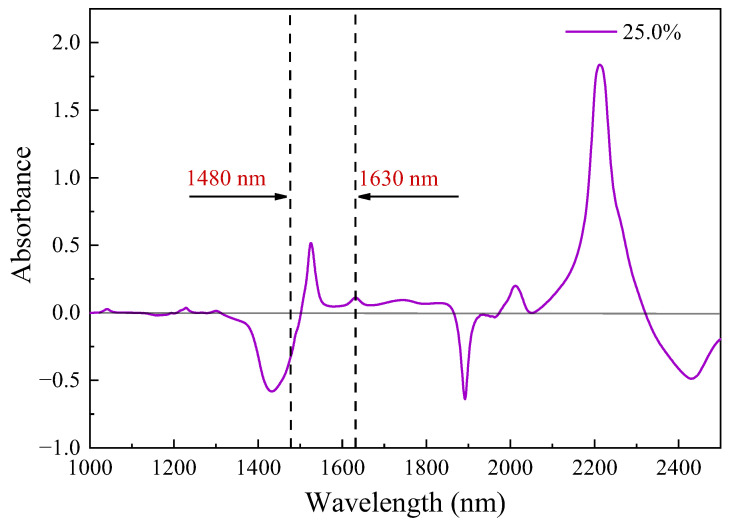
The near-infrared absorption spectrum of aqueous ammonia ranging from 1.0 µm to 2.5 µm.

The spectral information of the analyte of interest is one of the important parameters to guarantee measurement accuracy. However, unlike most gaseous molecules with a publicly accessible spectral database [48], as far as we know, the spectral parameters of aqueous ammonia are rarely reported in the near infrared range. Herein, capturing precise absorption information for aqueous ammonia is imperative, particularly its absorption peak position and broad spectral profile. A commercial FT-IR spectrometer (VERTEX 80v, Bruker, Billerica, MA, USA) was employed for laboratory calibration due to its exceptional advantages, including superior resolution, heightened sensitivity, excellent signal-to-noise ratio, remarkable repeatability, and extensive measurement range. During the measurement process, a custom glass cuvette was used as a liquid cell with an optical path of 1 mm. Pure water served as the initial background reference for further analysis, while 25% aqueous ammonia was utilized as a signal. The absorption spectrum was then calculated based on Equation (5), shown in Figure 6, within the near-infrared spectral range from 1.0 µm to 2.5 µm. The concentration is certified by the sample supplier according to ISO Guide 35.

**Figure 7:**
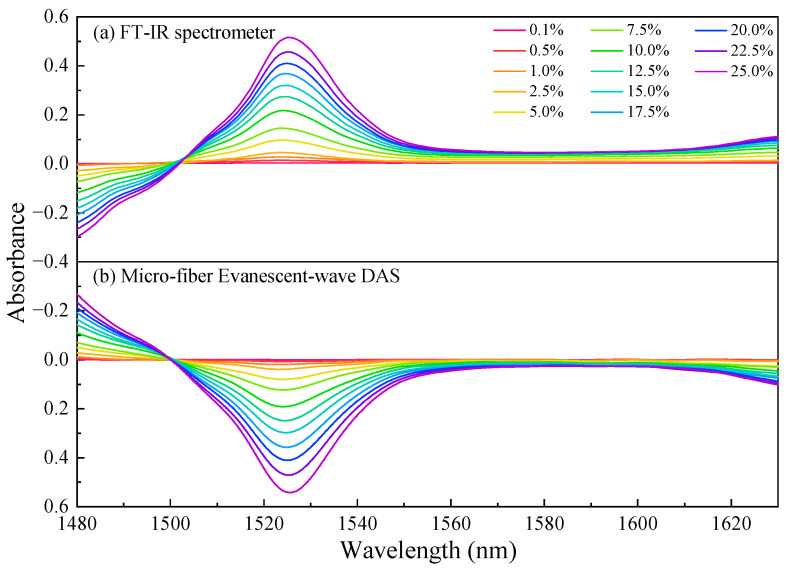
Absorption spectra of aqueous ammonia with varying concentrations using (**a**) the FT-IR spectrometer and (**b**) the silica micro-fiber evanescent-wave direct absorption sensor within the ECDL working range of 1480–1630 nm.

The absorption spectrum of ammonia in a liquid environment exhibits a broad spectrum, featuring positive absorption peaks at approximately 1525 nm, 2012 nm, and 2212 nm, as well as negative absorption peaks near 1433 nm, 1892 nm, and 2430 nm. The absorbance signal in Figure 6 is primarily a result of the combined absorption of the solvent (water molecules) and the solute (ammonia molecules). When the background absorption intensity exceeds that of aqueous ammonia, Equation (5) will yield a negative value, which is a normal occurrence and does not impact the analysis of experimental results. In order to ensure the accuracy of detection information, it is advisable to carefully select spectral bands characterized by strong absorption and optimize laser coverage at the forefront. Thus, we opt for utilizing an external cavity diode laser (TSL-550, Santec, Komaki, Japan) as the light source for our fiber evanescent-wave absorption sensing system due to its exceptional wavelength tuning range that precisely covers the aqueous ammonia absorption peak around 1525 nm. During the experiment, the laser, propagating through the micro-fiber, systematically scanned from 1480 nm to 1630 nm using a sawtooth wave pattern at a rate of 30 nm/s. The injection pump introduced aqueous ammonia samples of different concentrations (ranging from 0.1% to 25%) into the sensor at a stable low flow rate of 0.05 mL/min. Prior to each sample change, a thorough cleaning of the channel with ultrapure water was conducted, and measurements were taken for output power under flowing water conditions for subsequent data analysis. Finally, signal processing and analysis were performed by combining Equation (5). The measurement results of varying-concentration aqueous ammonia are shown in Figure 7.

**Figure 8:**
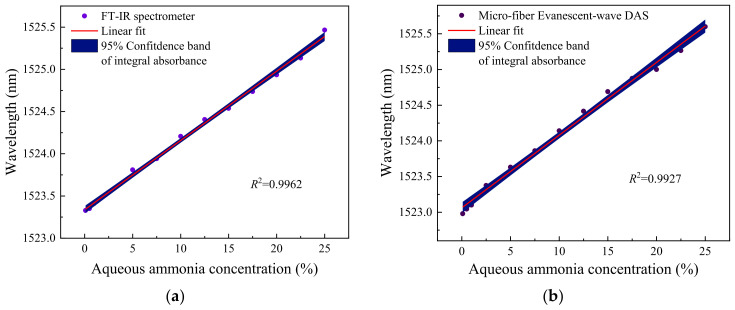
The relationship between the peak position and aqueous ammonia concentration in (**a**) the FT-IR spectrum and (**b**) the silica micro-fiber evanescent-wave direct absorption spectrum.

The two detection methods have good consistency in the spectra. It is of interest to observe a red shift phenomenon in both measurement methods, where the absorption peak position of aqueous ammonia tends to shift towards longer wavelengths with increasing concentration. This phenomenon may be attributed to the fact that the peak of the absorption signal primarily consists of the absorption contributions from water and ammonia molecules, representing the maximum point of their combined absorptions. As the concentration of ammonia molecules increases, it inversely correlates with the concentration of water molecules, resulting in corresponding increments and decrements in their respective absorption coefficients. Consequently, this inevitably leads to a shift in the absorption peak. Figure 8 shows the correlation between the peak wavelength and concentrations of aqueous ammonia for both methods. Linear regression analysis yields coefficients of determination (i.e., R-square values) of 0.9962 and 0.9927, respectively, indicating a strong linear correlation between the peak wavelength of the sensor and the concentration of the solution.

### 4.2. Performance Evaluation

**Figure 9:**
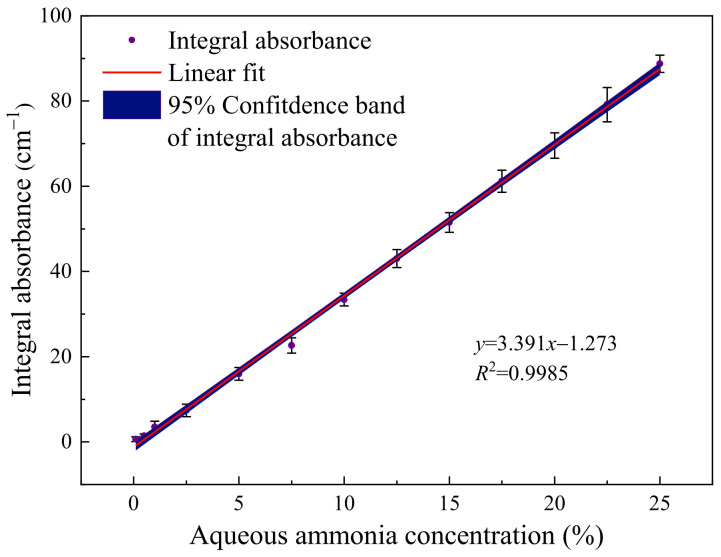
The measured integral absorbance as a function of aqueous ammonia concentration and error bars are magnified five times. The linearity calibration was performed by introducing samples of varying concentrations into the microchannel at a constant flow rate of 0.05 mL/min.

Equation (6) in the second section infers a linear relationship between the integral absorbance signal of the evanescent-wave direct absorption spectrum and the liquid concentration. Therefore, by detecting samples with different standard concentrations and establishing a linear equation for sensor calibration, the inversion of the measured liquid concentration can be realized. The corresponding relationship between the amplitude of the integral absorbance signal and the aqueous ammonia concentration is experimentally obtained with the results shown in Figure 9, and the vertical error bars (1-σ standard deviation) are obtained by evaluating the variation of the measurement results of 25 sets of experimental data, indicating good linearity with an R-square value of 0.9985 and a responsivity of ~3.391 cm^−1^/%.

**Figure 10:**
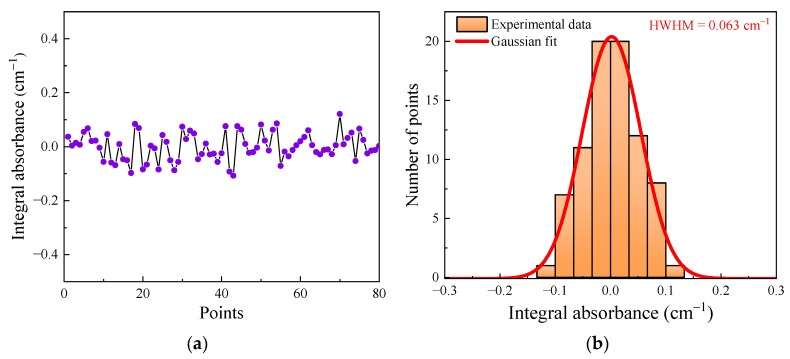
(**a**) The amplitude of the continuous measured integral absorbance, and (**b**) the Gaussian distribution of the 80 data points.

To evaluate the minimum detection limit (MDL) of this developed dissolved gas sensor, we record a continuous measurement of pure water background for about 15 min with a temporal resolution of 10 s. Figure 10a presents the recorded results, yielding a standard deviation of 0.050 cm^−1^. A corresponding MDL of 0.015% is determined using the analysis results in Figure 9. To access the precision of this dissolved gas sensor, a histogram plot of the measured data is shown in Figure 10b, which characterizes the noise distribution around the mean value of 0. A Gaussian fitting was performed, indicating a half width at half maximum (HWHM) of 0.063 cm^−1^. The precision is determined to be 0.018% with the HWHM.

**Figure 11:**
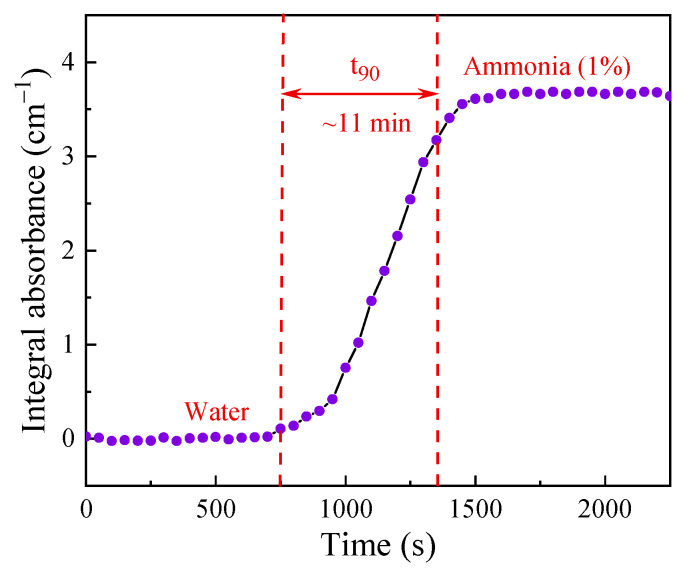
The response time (t_90_) of the system was measured at a flow rate of 0.05 mL/min, with the aqueous ammonia concentration changing from zero to 1%.

For optical fiber sensors, the response time is usually defined as the time that is required by the sensor to reach 90% of the maximum signal [49]. By changing the sample concentration from 0 (pure water) to 1%, we obtained the real-time integral absorbance of the sensor, as shown in Figure 11. The response time of the sensor is calculated to be about 11 min, i.e., t_90_ ≈ 11 min, proving itself as a more efficient measurement method compared to that of membrane separation technologies [21,24,50].

### 4.3. Discussion

By detecting the evanescent-wave absorption spectrum of aqueous ammonia using a simple micro-fiber (diameter, 2 µm; taper waist length, 5 mm) without any sensibilization treatment, we have proven the possibility of direct measurement of dissolved ammonia gas in water without separating the gaseous ammonia from the sample solution. After a preliminary test, the current sensor achieved a detection responsivity of 3.391 cm^−1^/%, a response time of about 11 min, and a minimum detection limit of 0.015%. It is of interest to discuss further performance improvement for wider aquatic applications.

In terms of the sensing unit, plating an analyte-sensitive layer on the fiber surface enables contact area enhancement with photons [51]. This modification would increase the absorption intensity of the analyte, indirectly improving both the MDL and absorption selectivity while preventing interference from other species. Moreover, incorporating Fiber Bragg Grating (FBG) at both ends of the sensing fiber to create a fiber Fabry–Pérot cavity is another effective alternative strategy. This approach would effectively extend the light–matter interaction path and further enhance the MDL. However, it is crucial to ensure good optical coupling efficiency for this method without sacrificing other performances. Additionally, decreasing the tapered waist diameter [40,52], choosing a stronger absorption peak, e.g., the absorption peak at 2200 nm in Figure 6, and combining optical fiber ring decay technology [53] are also possible strategies for sensitivity enhancement.

The response time of our sensor has been improved to a minute level, much faster compared to the traditional membrane separation technology. Sub-minute or even second-level response is still necessary in some scenarios where measuring the dynamic variation and distribution of dissolved gas is needed. Considering the negligible volume of the microchannel and the inlet tube (35 µL), the main factors limiting the response time are the injection rate and the volume of the buffer (*T*_time_ = *V*_volume_/*S*_rate_). An increased injection rate (*S*_rate_) would effectively promote the response time in a simple way. However, one must consider the possible micro-fiber deformation due to its extremely small waist diameter and excessive flow rate, which need a much more detailed test before real applications. The liquid content of the buffer (550 µL) also restricts the response time; when measuring samples of different concentrations, the sample of the former concentration needs to be replaced by the sample of the latter concentration through the buffer device, and response detection is carried out in the sensing area. The large buffer volume (550 µL) and slow injection rate (0.05 mL/min) contribute to the overall response time. Without the buffer, the ideal response time can even be several seconds, but at the cost of potential interference from external air bubbles. Therefore, carefully adjusting the flow rate and reducing the volume of the buffer are possible strategies in future investigations to improve the response time.

In the analysis of multi-component mixtures, absorption spectroscopy must account for the interference caused by overlapping absorption bands. To address this issue in our subsequent research, we will not only focus on modifying optical fiber but also incorporate frequency-resolved spectroscopic technologies in the micro-fiber platform [54] and decoupling techniques for further exploration. The powerful computing power of machine learning effectively distinguishes signals from noise and shows potential for solving absorption overlap problems [55,56].

## 5. Conclusions

In summary, we used a hydrogen flame to stretch silica single-mode fiber to obtain tapered fiber with a controllable waist diameter and length and successfully verified the feasibility of the evanescent-wave direct absorption spectrum of fiber without any modification in the liquid phase. Taking the dissolved ammonia nitrogen in water as the detection target, both the absorption lines and obvious red shifts are consistent with the results of a precision FT-IR spectrometer. Under normal temperature and pressure, the integral absorbance signal amplitude of the sensor has a good linear relationship with the standard aqueous ammonia concentration, the square of the correlation coefficient is 0.9985, and a responsivity of ~3.391 cm^−1^/%. In the presence of a liquid buffer, the response time is ~11 min, and the MDL is accessed at 0.015%. Several strategies are discussed to further enhance the performance of the current sensor system. Despite the present system’s relatively modest detection performance, we believe the technique of this study serves as a valuable candidate for future marine dissolved gas detection.

## Data Availability

The data that support the plots within this paper are available from the corresponding author on a request basis.
